# Inflammatory Pseudotumor Complicated by Recurrent Dislocations after Revision Total Hip Arthroplasty

**DOI:** 10.1155/2014/792781

**Published:** 2014-08-04

**Authors:** John Ryan Quinn, Jason Lee, Ran Schwarzkopf

**Affiliations:** Department of Orthopaedics, Joint Replacement Surgery, University of California, Irvine, 101 The City Drive South, Pavilion III, Building 29A, Orange, CA 92868, USA

## Abstract

A 71-year-old female with a history of right total hip arthroplasty presented with an enlarging pseudotumor. Pseudotumor is a known complication following metal-on-metal and metal-on-conventional polyethylene and metal-on-highly cross-linked polyethylene implants. Revision total hip arthroplasty following resection of pseudotumor has resulted in an increase in incidence of postoperative complications. Despite stable implants, these complications arise from the amount of soft tissue damage combined with the loss of tissue support around the resected hip. Our case is a clear example of a major complication, recurrent dislocation, following resection and revision surgery.

## 1. Introduction

Periprosthetic inflammatory masses or cysts are rare yet known complications of total hip arthroplasty (THA). They are commonly referred to as “inflammatory pseudotumors” [1]. The inflammatory component is derived from histological findings, which include periarticular tissue necrosis and perivascular lymphocytic inflammation also known as aseptic lymphocyte-dominated vasculitis-associated lesion (ALVAL) [2–6]. The recent revival of second-generation metal-on-metal (MoM) hip replacements, due to decreased wear rates, has been associated with the development of these inflammatory pseudotumors [7–9]. They have also been reported after metal-on-conventional polyethylene bearing THA [10–15]. A recent report showed formation of a pseudotumor with metal-on-highly cross-linked polyethylene bearing THA [16]. Pseudotumors cause a spectrum of clinical problems, ranging from an asymptomatic lesion to a large mass causing significant symptoms: pain, discomfort, or nerve palsy [17]. Resection of these pseudotumors is difficult and can lead to an increase in complication rates following revision total hip arthroplasty surgery. The recommendation for pseudotumor resection is to remove as early as possible in order to limit the amount of soft tissue damage [18, 19]. We present a severe, complex case, where the resection and revision THA resulted in significant anterior soft tissue damage and tissue loss leading to anterior instability facilitating recurrent dislocation.

## 2. Case Report

**Figure 1:**
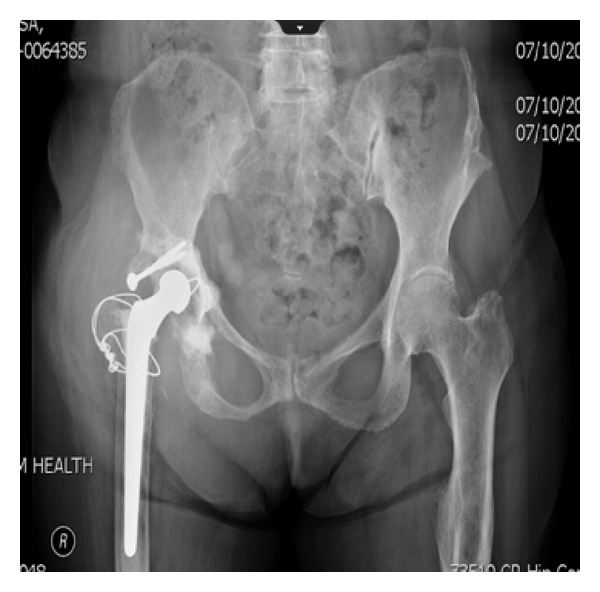
AP radiograph of pelvis revealing previous right total hip arthroplasty.

**Figure 2:**
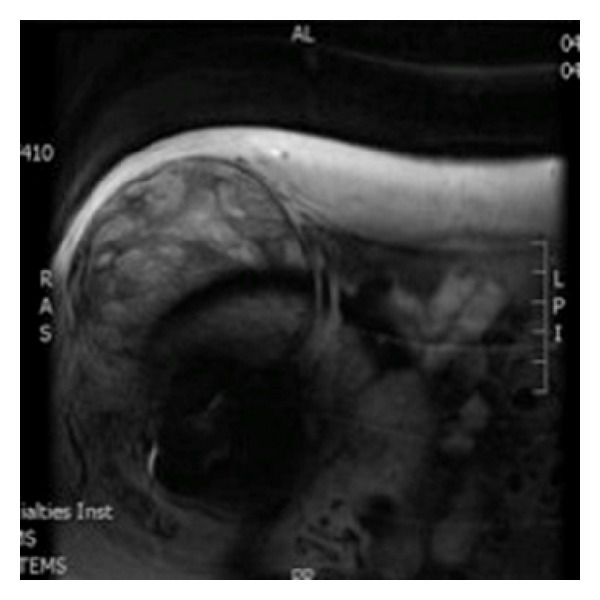
MRI showing 12.0 × 12.1 × 14.3 cm multilobulated soft tissue mass.

A 71-year-old female with a history of right THA in 1975, as well as a right total knee arthroplasty in 1992, presented to clinic with a chief complaint of an enlarging right hip soft tissue mass (Figure 1). The mass was initially noted 6 months previously and was evaluated at an outside clinic by MRI. The MRI revealed a 12.0 × 12.1 × 14.3 cm multilobulated soft tissue mass of the right hip region (Figure 2). She denied any pain, discomfort, paresthesia, or weakness. Her previous THA consisted of an old Charnley cemented components. Physical examination of the right hemipelvis and lower extremity revealed a firm, immobile, nontender mass measuring around 14.0 × 12.0 cm. Range of motion of the right hip was noted at 50 degrees of flexion, 5 degrees of internal rotation, 15 degrees of external rotation, and limited abduction. There was a difference in leg lengths on exam with the left leg being 1 cm shorter than the right.

**Figure 3:**
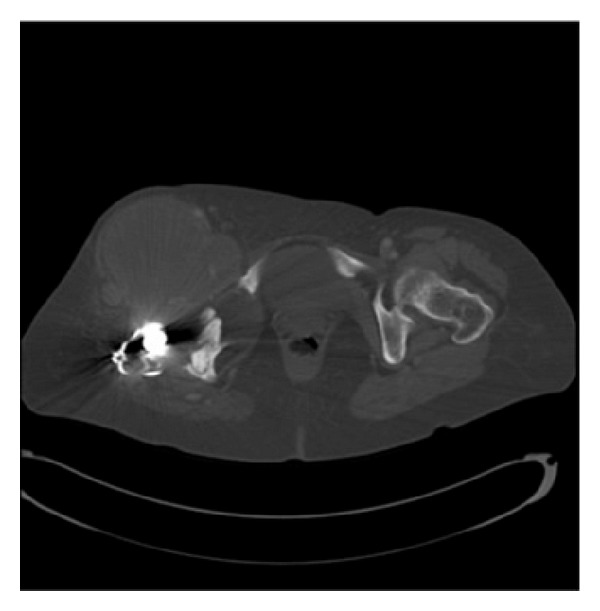
CT scan showing the mass originating from the right hip joint and extending predominantly anteriorly and medially.

Lab work was significant for an elevation of ESR and CRP at 46 mm/hr (ESR < 30 mm/hr) and 5.9 mg/dL (CRP < 0.7 mg/dL), respectively. White blood cell count was within normal limits. Radiographic images revealed signs of both acetabular and femoral loosening with previous wires from a greater trochanter osteotomy. The radiograph also revealed decreased bone mineralization and severe osteolysis especially in the proximal femur with complete resorption of the greater trochanter. CT scan with contrast showed a 15.0 × 7.5 × 9.6 cm mass originating from the right hip joint and extending predominantly anteriorly and medially with mass effect (Figure 3). Core needle biopsy showed no malignant cells. It consisted of mostly blood and fragments of fibrous tissue, most commonly associated with pseudotumor.

**Figure 4:**
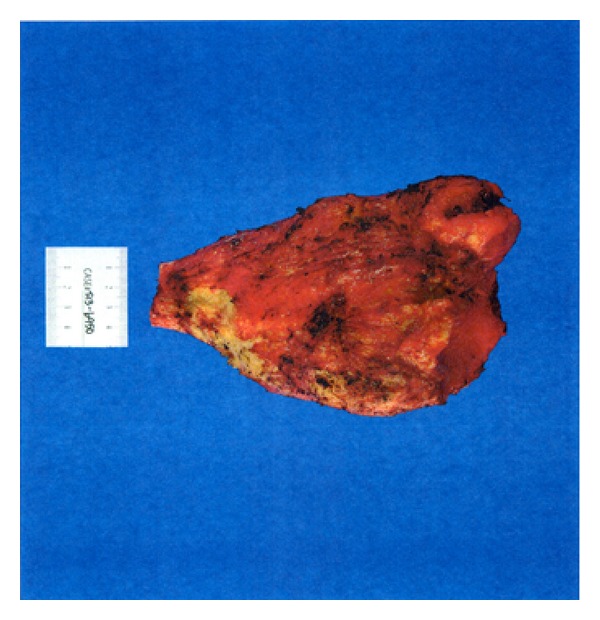
Pseudotumor status after resection.

**Figure 5:**
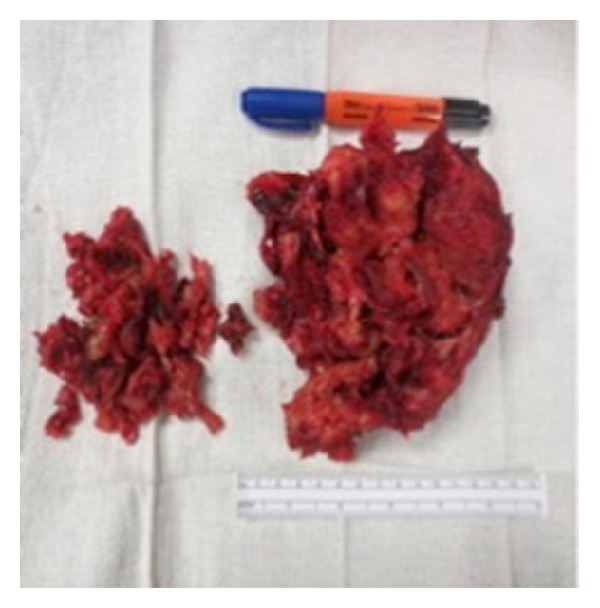
Gross image of the dissected pseudotumor.

**Figure 6:**
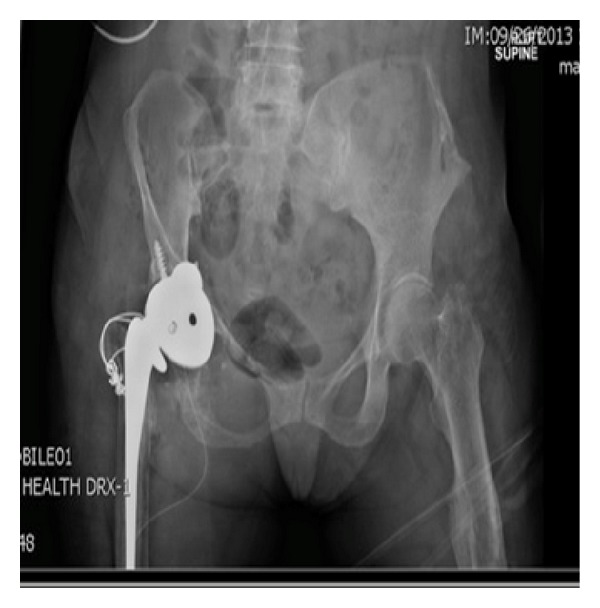
Revision total hip arthroplasty complicated by anterior dislocation.

**Figure 7:**
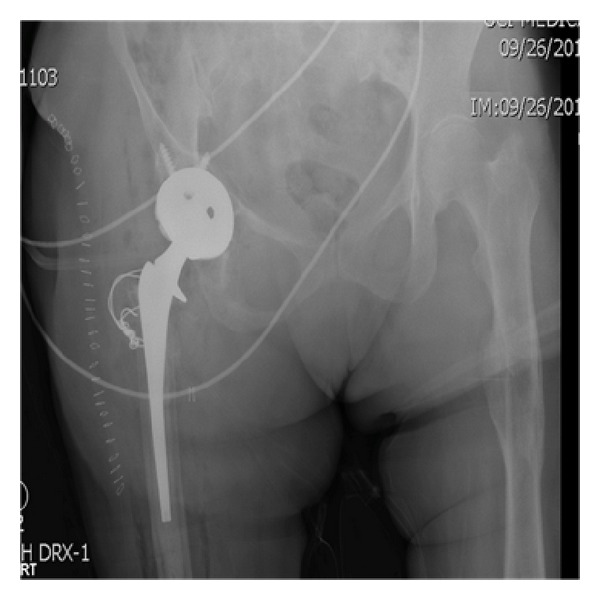
Postoperative radiograph status after reduction of dislocated hip.

The diagnosis of pseudotumor resulted in resection with right revision THA. A multispecialty team comprised of a fellowship trained orthopaedic tumor surgeon and an adult reconstruction fellowship trained orthopaedic surgeon performed the surgery. A direct anterior approach was utilized due to the location of the tumor and the patient's previous scar, and the incision was taken down through the scar tissue and the fascia. The tumor was located bulging through the tensor fascia lata, and the capsule was dissected circumferentially. It was tracked down through the superior pubic ramus and down toward the ischium. The tumor was removed all the way down to the anterior rim of the acetabulum leaving a large gap between skin and acetabulum (Figures 4 and 5). The subsequent procedure, revision THA, was warranted secondary to the severe osteolysis from polyethylene wear and aseptic loosening of the femoral component. After resection of the tumor, inspection of the acetabulum showed a loosened acetabular shell with significant anterior wall and anterior column bone loss. The acetabulum was revised and a trabecular metal revision shell (Trabecular Metal Acetabulum Revision System; Zimmer, Warsaw, IN) was press fit in a position of maximum bony contact and secured with multiple screws. A highly cross-linked polyethylene liner was cemented in 45 degrees of abduction and 20 degrees of anteversion. The stem was removed from the cement mantle without difficulty. Due to the low bone quality, the previous cement mantle, and the location of the total knee femoral stem, it was decided that the only feasible option to secure a new femoral implant would be to cement a new femoral stem into the existing cement mantle. Due to the instability anteriorly during trial reduction, the stem was cemented in neutral version (Synergy; Smith and Nephew, Memphis, TN). The hip had good range of motion with flexion to 100 degrees and flexion to 90 degrees with more than 80 degrees internal rotation. Impingement was noted on extreme extension, external rotation, and adduction, but it was determined that the patient would not achieve this extreme range of motion. Radiographic evaluation in the recovery room showed an acute hip dislocation (Figure 6). Her immediate postoperative care was complicated by anterior dislocation. Her impingement of the femoral neck implant on the posterior aspect of the acetabular cup resulted in dislocation due to lack of any anterior soft tissue restraint. The patient returned to the operating room and the dislocated joint was visualized. It was decided to revise the cemented acetabular liner and place it in 45 degrees abduction and neutral version. Her range of motion was tested again and she was found to be stable in extension, external rotation, and adduction as well as flexion greater than 100 degrees and flexion of 90 degrees with 80-degree internal rotation. Postoperative radiographs were taken to verify position of the components (Figure 7).

**Figure 8:**
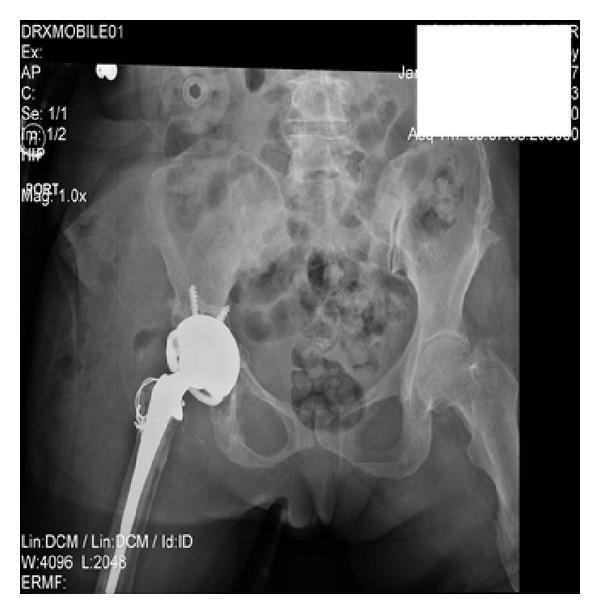
Re-revision surgery status after constrained liner.

Follow-up two and a half weeks later revealed recurrent dislocation. She was taken to the emergency department where closed reduction was attempted unsuccessfully. The patient was taken to the OR where again the significant anterior soft tissue loss was noted facilitating anterior dislocation. It was decided to place a constrained liner to prevent further dislocation. Range of motion was tested and her hip was stable (Figure 8).

**Figure 9:**
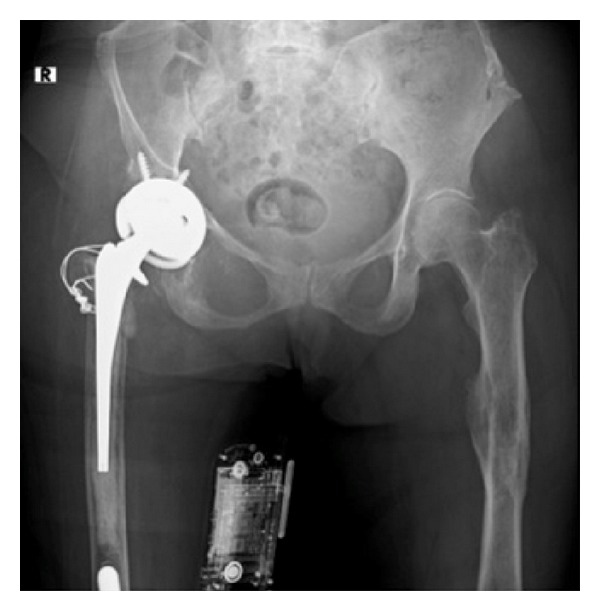
3 months after operation.

**Figure 10:**
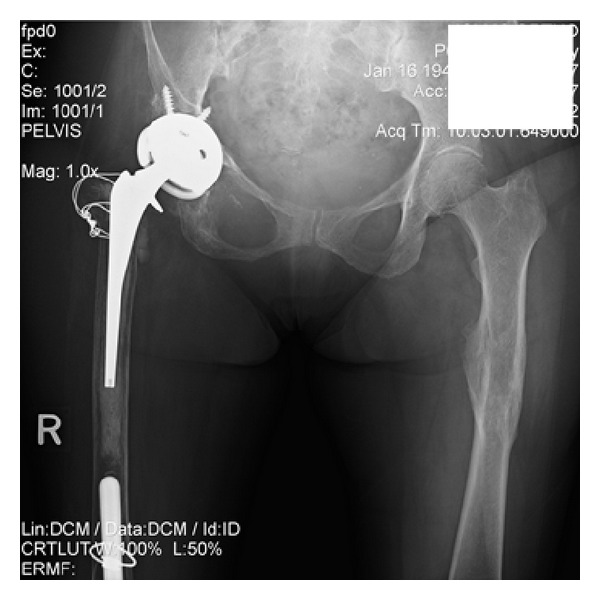
5 months after operation.

**Figure 11:**
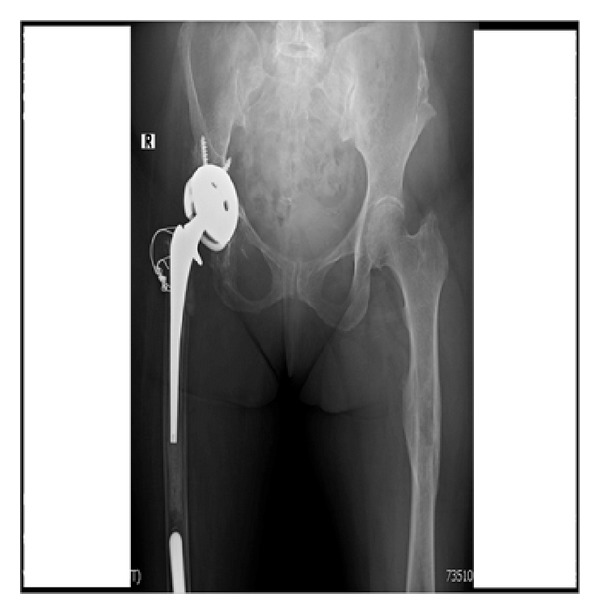
8 months after operation.

At the 3-month follow-up the patient was doing well and radiographic images revealed a stable implant (Figure 9). She continues to progress and is currently full weight bearing with no pain. Follow-up radiographs continue to reveal a stable implant (Figures 10 and 11).

## 3. Discussion

Inflammatory pseudotumors have complicated all bearings of THA including MoM, metal-on-conventional polyethylene, and metal-on-highly cross-linked polyethylene [10–16]. The reported incidence rate of pseudotumor formation in MoM by Pandit et al. is one percent of THAs within 5 years [16]. No incidence rates for the other bearings have been reported in the literature. This same group calculated the rate of revision during pseudotumor resection focusing on specific risk factors: age, gender, unilateral/bilateral THA, components, and histological features. They concluded that revision rate for pseudotumors in men under 40 years at 8 years was 0.5 percent compared to women with a rate of 13.1 percent at 6 years [16, 17]. They identified four specific risk factors that were associated with an increase in revision rate: female gender, age under 40, small components (<46 mm in females, <50 mm in males), and dysplasia [17]. Female gender, age under 40, small components, and dysplasia increase the failure rate by 8.2, 3.3, 6.6, and 3 times, respectively [17]. Other studies have reported revision rates as high as 25 percent in women under 40 years of age and 5 percent in women over 40 years [16]. Our patient had all risk factors at the time of revision surgery: female, under 40 years when she received THA, smaller components (<46 mm for female), and demonstrated dysplasia. She was at a high risk of revision after pseudotumor resection.

The outcome of revision surgery for pseudotumor is considerably worse than the outcome of other THAs [18]. Grammatopoulos et al. described a major complication rate of 50 percent after revision surgery following pseudotumor resection related to hip resurfacing [18]. The major complication rate of 50 percent is considerably higher than the rate of 14 percent after revision for other causes [18]. This study identified 53 hips that had undergone MoM hip resurfacing that required revision. Of these revised, 16 were due to pseudotumor. These surgeries tended to be more complex secondary to the soft tissue destruction. Of the 16 hip revisions, 12 required blood transfusion postoperatively, and 8 experienced major complications [18]. Three of the revision hips experienced femoral nerve palsy, two had loosening of the acetabular component within two years, and three dealt with dislocation, two of which were recurrent [18]. All of these dislocations required femoral and acetabular component revision. Of interest, three out of the five recorded that underwent re-revision had evidence of recurrence of pseudotumor [18]. This study also illustrated the complexity of pseudotumor revision cases by measuring operating room time, functional outcome by the Oxford hip score (OHS), and the amount of activity by the University of California Los Angeles (UCLA) activity score [18]. Pseudotumor revisions showed a drastically increased operating room time, OHS score was significantly worse in pseudotumor revisions (20.9 versus 40.2 and 37.8 in other groups), and UCLA activity score revealed a drop in activity [18]. Again, the outcome of revision for pseudotumor is poor. In fact, one-third of the patients undergoing revision for pseudotumor will have future re-revision surgeries [18]. Our case was a clear example of the complexity of pseudotumor revision. Revision surgery was complicated by recurrent dislocation requiring re-revision surgery with a constrained acetabular liner being placed. Our patient's surgery resulted in a loss of anterior capsule and musculature limiting her anterior physical stability to basically skin. This large gap was an enormous loss of stability. A constrained liner was placed to provide anterior stability decreasing her risk of dislocation. Aggressive early intervention is warranted in pseudotumor cases in order to decrease the amount of soft tissue loss leading to better outcomes. It is imperative to consider the risk factors associated with an increased rate of revision as early intervention may provide a more stable hip. Our case further supports the research done by Glyn-Jones et al. as she had all risk factors associated with a higher risk of revision [17]. Our case was complicated not only by pseudotumor, complete resorption of the greater trochanter, and soft tissue loss but also by a previous total knee arthroplasty limiting our stem selection. The treatment options were limited, and ultimately a constrained liner was placed. Another feasible treatment option is covering the large defect anterior to the capsule with a transverse rectus abdominis pedicle flap [18]. The flap could be placed over the anterior capsule providing that anterior stability needed to prevent complications such as recurrent dislocation. This treatment option could have benefitted our patient because the pseudotumor resection left a limited barrier of only skin and small musculature fibers over the anterior portion of her hip joint. The outcome of such surgery has never been reported and would need further verification of the success rate.

Overall, pseudotumor resection and revision THA is a complicated case with an increased incidence of postoperative complications. It is important to recognize patients that are at an increased risk of revision after pseudotumor resection because early aggressive intervention can be beneficial. If a large amount of soft tissue is destroyed or lost, then aggressive treatment options such as a constrained liner or flap could be utilized to try to decrease the fifty percent major complication rate or 33 percent re-revision surgeries required.
